# Control of measles in a juvenile custodial setting in the wake of the recent US outbreak

**DOI:** 10.11604/pamj.supp.2020.35.1.20172

**Published:** 2020-01-03

**Authors:** Sadhana Dharmapuri, Karen Simpson, Kenneth Soyemi

**Affiliations:** 1 Department of Pediatrics, John H Stroger Hospital of Cook County, Chicago, Illinois, United States; 2 Cermark Health Services @Cook County Juvenile Temporary Detention Center, Chicago, Illinois, United States

**Keywords:** Juvenile, custodial, setting, measles, prevention

## Abstract

The recent US measles outbreak is the largest since 1992. It is just a matter of time before measles is introduced into a juvenile custodial setting. Are we prepared? Should we be prepared? This short article addresses steps institutional settings should take to prevent the spread of measles in a contained setting.

## Brief

Measles is a contagious disease with a high rate of transmission in vulnerable populations. When introduced into a closed custodial setting such as jails, prisons, or juvenile detention centers, the number of potential new infections can rise exponentially depending on the immunization status of the inmates or residents. The US is experiencing the largest outbreak since 1992; according to the Centers for Disease Control and Prevention (CDC), over 1,000 infections have been reported from 28 states in 2019 [1]. Measles has a high reproductive number, meaning one infected person or resident has the potential to infect between 17-20 susceptible persons. Because of high infectivity, closed settings have to be prepared to rapidly identify, isolate and vaccinate vulnerable residents. We aim to address juvenile custodial setting outbreak prevention and immunity monitoring during the current high alert measles situation in the US measles can be introduced into a closed setting from external sources such as new detainees entering into the facility and staff, visitors, contractors or vendors working in or visiting the facility. Screening staff and residents for immunity, is cost effective and necessary to prevent measles introduction. The goal of screening will be to identify potential vulnerable residents and staff and in the event of an outbreak exclude them from work or isolate them to prevent disease transmission. Steps to follow in the event of an outbreak in a closed setting include the following: 1) Immediately isolate the suspected resident / inmate and implement contact precautions and post exposure prophylaxis (PEP). 2) Confirm diagnosis using clinical, and laboratory parameters see Table 1 for definitions. 3) Call your local health department upon suspicion; confirm disease using clinical and laboratory parameters (see definitions in Table 1). 4) Staff, visitors, and vendors exposed to measles who cannot readily show that they have evidence of immunity against measles should be offered PEP or be excluded from the facility. 5) To provide protection or modify the clinical course among susceptible residents/inmates, staff or vendors, either administer the MMR vaccine within 72 hours of initial exposure or immunoglobulin (IG) within six days of exposure. Do not administer the MMR vaccine and IG simultaneously, as this practice invalidates the vaccine. 6) If the MMR vaccine is not administered within 72 hours as PEP, the vaccine should still be offered in order to offer protection from any future exposures. Those who receive the MMR vaccine or IG as PEP should be monitored for signs and symptoms consistent with measles for at least one incubation period (7-21 days). 7) Infected inmates or residents should be isolated for four days after they develop a rash. 8) Work on logistics such as getting security clearance to enable local health department staff to enter the facility. 9) Stop the transfer of inmates or residents in and out of the custodial facility to reduce the risk of spreading measles to other parts of the facility.

**Table 1:** Case definition and epidemiological classification^s^

Outbreak	Measles outbreaks are defined as three or more cases
Clinical description	An acute illness characterized by: Generalized, maculopapular rash lasting ≥3 days; and Temperature ≥101°F or 38.3°C; and Cough, coryza, or conjunctivitis.
Probable	In the absence of a more likely diagnosis, an illness that meets the clinical description with: No epidemiologic linkage to a laboratory-confirmed measles case; and Noncontributory or no measles laboratory testing.
Confirmed	An acute febrile rash illness with: Isolation of measles virus from a clinical specimen; or Detection of measles-virus specific nucleic acid from a clinical specimen using polymerase chain reaction; or IgG seroconversion or a significant rise in measles immunoglobulin G antibody using any evaluated and validated method; or A positive serologic test for measles immunoglobulin M antibody; or Direct epidemiologic linkage to a case confirmed by one of the methods above.
Internationally imported case	An internationally imported case is defined as a case in which measles results from exposure to measles virus outside the United States as evidenced by at least some of the exposure period (7–21 days before rash onset) occurring outside the United States and rash onset occurring within 21 days of entering the United States and there is no known exposure to measles in the U.S. during that time. All other cases are considered U.S.-acquired.
U.S.-acquired case	The patient had not been outside the United States during the 21 days before rash onset or was known to have been exposed to measles within the U.S.
Import-linked case	Any case in a chain of transmission that is epidemiologically linked to an internationally imported case
Imported-virus case	a case for which an epidemiologic link to an internationally imported case was not identified, but for which viral genetic evidence indicates an imported measles genotype
Endemic case	a case for which epidemiological or virological evidence indicates an endemic chain of transmission. Endemic transmission is defined as a chain of measles virus transmission that is continuous for ≥12 months within the United States.
Unknown source case	a case for which an epidemiological or virological link to importation or to endemic transmission within the U.S. cannot be established after a thorough investigation.

Internationally imported, import-linked, and imported-virus cases are considered collectively to be import-associated cases

a council for state and territorial epidemiologist case definitions

According to the bureau of prisons immunization guideline, during a measles outbreak in an adult custodial setting, it is recommended that one dose of Measles-mumps-rubella (MMR) vaccine be given to persons identified to be at risk and to those who have no evidence of immunity to measles within 72 hours of exposure [2]. As of 2016, there are approximately 1,772 juvenile facilities of which 662 are detention centers. Annually, the detention centers remand an estimate average of 15,000 residents. To the best of our knowledge, there has been no report of a measles outbreak in a juvenile custodial setting; search of databases revealed a few reported measles outbreak cases in adult custodial settings [3–6]. The receipt of 2 or more MMR vaccines in the US is more than 90 percent among US adolescents aged 13 to 17 years across all ethnic groups, metropolitan statistical area, rural and non-rural counties and states, according to the national immunization survey [7]. The MMR vaccine update trend in the birth cohorts continues to remain high from 2008 through 2017, and we postulate that the high MMR vaccine rate might be a contributing factor to the paucity of the measles outbreak in juvenile custodial settings. Previous prison outbreak mitigation efforts demonstrated that mass vaccination following an outbreak is not always likely to prevent new infections among susceptible individuals; favorable mitigating factors include implementing opt-out testing, vaccination, and requiring full immunization of staff, contractors, and vendors [5].

## Competing interests

The authors declare no competing insterests.
